# Seasonal Rainfall and Runoff Promote Coral Disease on an Inshore Reef

**DOI:** 10.1371/journal.pone.0016893

**Published:** 2011-02-10

**Authors:** Jessica Haapkylä, Richard K. F. Unsworth, Mike Flavell, David G. Bourne, Britta Schaffelke, Bette L. Willis

**Affiliations:** 1 ARC Centre of Excellence for Coral Reef Studies, School of Marine and Tropical Biology, James Cook University, Townsville, Australia; 2 Australian Institute of Marine Science, Townsville, Australia; 3 Biological Sciences Research Unit, University of Glamorgan, Pontypridd, United Kingdom; Institute of Marine Research, Norway

## Abstract

**Background:**

Declining water quality coupled with the effects of climate change are rapidly increasing coral diseases on reefs worldwide, although links between coral diseases and environmental parameters remain poorly understood. This is the first study to document a correlation between coral disease and water quality on an inshore reef.

**Methodology/Principal Findings:**

The temporal dynamics of the coral disease atramentous necrosis (AN) was investigated over two years within inshore populations of *Montipora aequituberculata* in the central Great Barrier Reef, in relation to rainfall, salinity, temperature, water column chlorophyll *a*, suspended solids, sedimentation, dissolved organic carbon, and particulate nitrogen, phosphorus and organic carbon. Overall, mean AN prevalence was 10-fold greater during summer wet seasons than winter dry seasons. A 2.5-fold greater mean disease abundance was detected during the summer of 2009 (44 ± SE 6.7 diseased colonies per 25 m^2^), when rainfall was 1.6-fold greater than in the summer of 2008. Two water quality parameters explained 67% of the variance in monthly disease prevalence in a Partial Least Squares regression analysis; disease abundance was negatively correlated with salinity (R2 = −0.6) but positively correlated with water column particulate organic carbon concentration (R2 = 0.32). Seasonal temperature patterns were also positively correlated with disease abundance, but explained only a small portion of the variance.

**Conclusions/Significance:**

The results suggest that rainfall and associated runoff may facilitate seasonal disease outbreaks, potentially by reducing host fitness or by increasing pathogen virulence due to higher availability of nutrients and organic matter. In the future, rainfall and seawater temperatures are likely to increase due to climate change which may lead to decreased health of inshore reefs.

## Introduction

Disease has emerged as a significant threat to wildlife populations in recent decades [e.g.1], [ 2]. A recent review highlights the substantial role that environmental nutrient enrichment has played in contributing to patterns of emerging human and wildlife diseases and the urgent need for studies to understand linkages, particularly in light of ongoing intensification of global nutrient cycles [3]. The current understanding of marine diseases is poor in comparison to knowledge of human, agricultural and terrestrial wildlife diseases [4]. It appears that epidemiological theories developed for terrestrial diseases may not translate well to marine ecosystems [4], [5]. For example, diseases appear to spread more rapidly in comparatively open oceanic ecosystems [6] and marine pathogens are more diverse taxonomically and in their life histories [5]. Thus, marine case studies that advance understanding of potential links between nutrient enrichment and marine diseases are critical if management tools for the long-term conservation of marine wildlife are to be effective.

Coral reefs are increasingly threatened by changes in water quality from terrestrial runoff [7], climate change [8], [9] and over-exploitation [10], [11]. Coral bleaching and disease have emerged as dominant drivers of coral population declines on coral reefs, particularly as oceans have warmed in the past few decades [12]. Current research supports a connection between a warming climate and increasing incidence of disease in corals [12], [13], [14]. For example, warm temperatures and high coral cover have been linked to increased abundance of white syndromes on the Great Barrier Reef (GBR) [14] and progression rates of black band disease were higher in the austral summer [15], [16]. However, links to most other anthropogenic disturbances are less clear [17].

Although the mechanisms are unknown, outbreaks of disease on some coral reefs have been correlated with increases in nutrient runoff [18], [19]. In the Philippines, a higher prevalence of growth anomalies and *Porites* ulcerative white spot disease was found near a sewage outfall [20], and white pox has also been linked to sewage inputs in the Caribbean [21]. Field experiments in the Caribbean have demonstrated that moderate increases in dissolved inorganic nutrient concentrations can substantially increase the severity of aspergillosis and yellow blotch diseases [22] and the prevalence of aspergillosis [18]. In other studies, nutrient exposure resulted in increased progression rates of black band disease, with nutrients thought to reduce the coral host's ability to counteract infection by pathogenic micro-organisms [23]. Experiments on the impacts of organic carbon on microbiota suggested that the mechanism may be indirect with elevated nutrients increasing the production of organic carbon (through primary production), which in turn leads to an increased growth rate of microbes living in the corals' mucus layer and a disruption of the balance between corals and their associated microbiota [24].

Terrestrial runoff to the inshore GBR is mainly delivered in short-lived flood events during the 5-month summer wet season [25], often forming distinct flood plumes in the coastal zone that sometimes reach far out into the GBR lagoon [26]. Elevated concentrations of nutrients, suspended sediments and pesticides, caused by changes in land use over the past 200 years of European settlement, are now potentially affecting the health of coastal and inshore ecosystems [25], [27]–[29]. In particular, sediment loads to the GBR have increased four to five-fold in this period [30], and five to ten-fold in some catchments [31]. Moreover, the area of the GBR affected by sediment inputs is increasing substantially as a result of changing land management practices, to the point where fine terrestrial sediment is reaching mid-shelf reefs for the first time in their geological history [30]. Sediments settling on corals may increase disease prevalence indirectly through increased stress and energy expenditure required to remove sediments [28], which could make them more susceptible to infections by microbial pathogens, and/or directly if sediments act as disease reservoirs [23].

Atramentous necrosis (AN) is one of the few coral diseases with high prevalence values on coastal GBR reefs (B. Willis and C. Page, pers. comm. 2008). AN was first observed in December 2001 on Magnetic Island, an inshore reef of the Central GBR [32], although subsequently also observed on reefs in both the northern and southern GBR (B. Willis and C. Page, pers. comm. 2008). In March 2002, a peak in AN causing significant mortality within Magnetic Island populations of the plate-like coral *Montipora aequituberculata* was observed during a thermal mass-bleaching event [32]. However, increased prevalence of AN was documented in spring (temperature <24.5°C), well before typical summer temperatures were reached [33], suggesting that temperature may not be the only environmental factor driving the occurrence of this disease.

AN progresses through four distinct stages: Stage 1 lesions are small (1–2 cm diameter) areas of bleached but intact tissue; Stage 2 lesions are white skeleton devoid of tissue; Stage 3 lesions are covered with a white bacterial film; and in Stage 4, a black, sulphurous deposit accumulates under the white film [33] likely the result of opportunistic secondary microbial community [34].

This is the first study to investigate a possible connection between the seasonal dynamics of a coral disease and parameters associated with water quality on the GBR. The aims of the present study were to (i) document seasonal dynamics of AN and nine seasonally varying environmental parameters, and (ii) analyse relationships between disease prevalence and these parameters to identify potential environmental drivers of AN within populations of the coral *Montipora aequituberculata* on an inshore GBR reef.

## Results

### (a) Dynamics of atramentous necrosis

**Figure 1:**
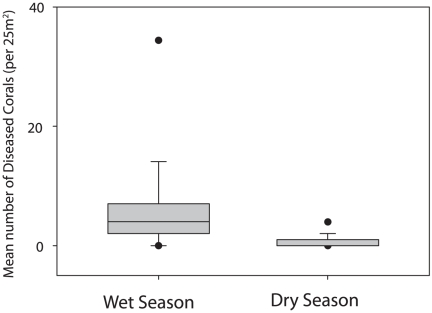
A box plot illustrating the distribution of the mean numbers of diseased corals between two seasons. Vertical bars illustrate standard deviations and horizontal bars medians. Black dots represent the 95 percentiles.

**Figure 2:**
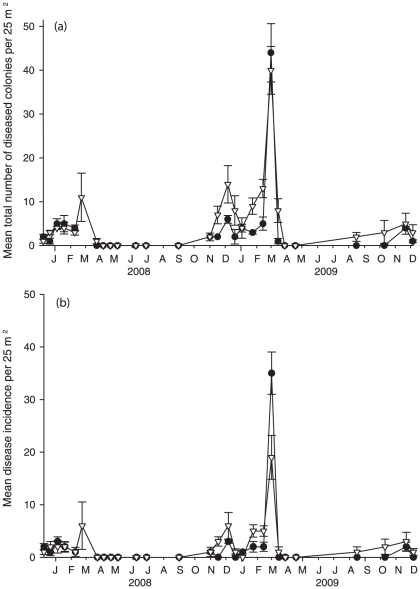
Mean number of diseased corals. (**a**) Mean number of corals demonstrating signs of atramentous necrosis (AN) per 25 m^2^ during the two-year study. The highest numbers were found in February 2009 in Geoffrey Bay (GB). (**b**) Mean number of new infections (i.e. incidence) of AN per 25 m^2^ during the two-year survey. The highest numbers were found in February 2009 in GB. (GB = dark circles, NB = white triangles).

**Figure 3:**
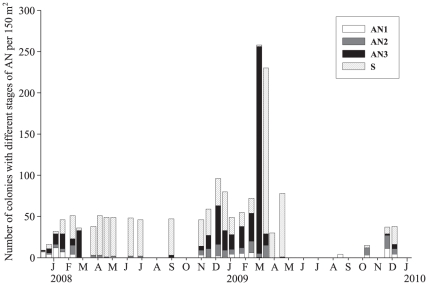
Disease stages in time. Disease stages in time per 150 m^2^ (AN1 = first stage of atramentous necrosis (AN) characterised by a small (1–2 cm diameter) initial area of bleached but intact tissue; AN2 = a lesion of white skeleton devoid of tissue; AN3 =  lesions covered with a white bacterial film and a black, sulphurous-smelling deposit, subsequently accumulating under the white film; S =  disease progression stopped). The third stage was most common during the summer disease peak whereas the disease stopped in winter.

At the two study sites (Nelly and Geoffrey Bays, Magnetic Island), a total of 379 colonies of *Montipora aequituberculata* showing signs of atramentous necrosis (AN) were tagged during the two-year study. The mean number of diseased corals was clearly higher in the wet season than in the dry season (Fig. 1). Highest values of both the mean number of AN cases and new disease cases (incidence) were measured in the end of February in both 2008 and 2009 (Fig. 2a,b), although the disease peak was four-fold greater in 2009. In 2009, the mean (±SE) number of diseased colonies was 44±6.67 colonies per 25 m^2^ in Geoffrey Bay (GB) and 40±5.46 colonies per 25 m^2^ in Nelly Bay (NB), whereas in 2008, 11±5.51 colonies were infected per 25 m^2^ in NB (Fig. 2a). The mean (±SE) incidence (i.e. number of new infections) was also higher in 2009, with 35±4.04 new infections per 25 m^2^ in GB and 19±4.18 per 25 m^2^ in NB, compared to only 6±4.51 new cases in NB in 2008 (Fig. 2b). Disease abundance decreased to 0–2 cases per 150 m^2^ in winter and re-appeared from November onwards in both years with 2^nd^ and 3^rd^ stages of AN (AN2 and AN3, respectively) being most common between January and March (Fig. 3).

### (b) Environmental conditions at the study sites

**Figure 4:**
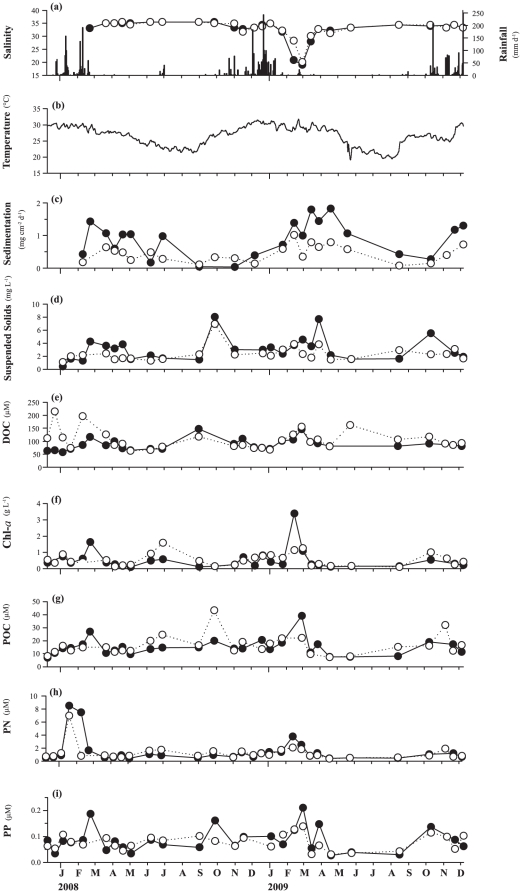
Temporal patterns in environmental variables. Temporal patterns in (a) salinity and rainfall, (b) daily mean sea water temperature combining temperatures from both bays, (c) ash-free dry weight of sediment (AFDW), (d) suspended solids (SS), (e) dissolved organic carbon (DOC), (f) chlorophyll *a* (chl-*a*), (g) particulate organic carbon (POC), (h) particulate nitrogen (PN), and (i) particulate phosphorus (PP) during the two-year study in Nelly and Geoffrey Bays. Values represent means of two samples at each study site. (NB = dark circles, GB = white circles).

The highest values for both mean abundance of diseased colonies and mean disease incidence corresponded with the highest values in all of the environmental parameters investigated except for salinity, for which the lowest values were recorded at the disease peak (Fig. 2 and 4). An increasing trend in environmental parameters (decreasing for salinity) was observed preceding the disease outbreaks in both years, with values tending to be higher in 2009 than in 2008. The summer of 2009 was the wettest in 10 years in the Townsville region, with a total rainfall of 1901.6 mm compared to 1187 mm in 2008. A dramatic (∼40%) decrease in salinity over four weeks was observed in 2009 prior to the disease outbreak (from 31.7 to 19.0 in NB, and from 32.3 to 20.1 in GB). Salinity data prior to the 2008 outbreak are lacking because measurements for this study started in February 2008. Sedimentation was very seasonal, with higher values during summer rain events, especially in 2009. Mean water temperatures increased by only 0.3°C (to 30.5°C) in the month prior to the disease outbreak in 2008, whereas temperatures increased by 1.7°C in the month prior to the 2009 outbreak and reached 31.7°C. The highest value of particulate nitrogen (PN) was observed one month prior to the 2008 outbreak, with lower and more even distribution of recorded values in 2009. Particulate phosphorus (PP) showed 10-fold higher values two weeks prior to the 2009 outbreak and particulate organic carbon (POC) values were higher in 2009 than in 2008 (Fig. 4, Table 1).

**Table 1:** Values for environmental parameters in 2008 and 2009.

Environmental parameter	1 month prior to dis. peak 2008		2 wks prior to dis. peak 2008		Disease peak 2008		1 month prior to dis. peak 2009		2 wks prior to dis. peak 2009		Disease peak 2009	
	NB	GB	NB	GB	NB	GB	NB	GB	NB	GB	NB	GB
**Salinity**					33.1		31.7	32.2	20.8	28.3	19.0	20.1
**Temp (°C)**	30.2		29.9		30.5		29.8		28.5		31.7	
**Rainfall (mm) Dec-Apr**				1187.0						1901.6		
**Sedimentation** **(ash-free dry weight mg cm^−2^ day^−1^)**			0.4	0.2	1.0		0.7	0.6	1.4	1.0	1.8	0.8
**Suspended solids** **(mg L^−1^)**	1.6	2.0	1.3	2.1	4.3		2.4	3.0	3.7	3.8	4.5	2.4
**Dissolved Organic Carbon (µM C)**	70.2	74.5	85.1	196.4	116.09		101.7	103.4	106.5	126.5	146.1	156.2
**Chlorophyll-** ***a*** **(µg L^−1^)**	0.4	0.4	0.6	0.4	1.6		0.3	0.7	3.4	1.1	1.1	1.3
**Particulate Organic Carbon (µM C)**	14.4	12.4	17.2	14.8	26.9		18.5	21.8			39.8	22.2
**Particulate Nitrogen** **(µM N)**	8.5	7.0	7.5	0.8	1.6		1.4	1.7	3.79	2.07	2.5	1.8
**Particulate Phosphorus (µM P)**	0.1	0.1	0.1	0.1	0.2		0.1	0.1	0.1	0.1	0.2	0.1

Values for the ten environmental parameters measured one month and two weeks prior to and during the disease outbreaks of 2008 and 2009. Values represent means of two samples at each study site (NB = Nelly Bay, GB = Geoffrey Bay).

**Figure 5:**
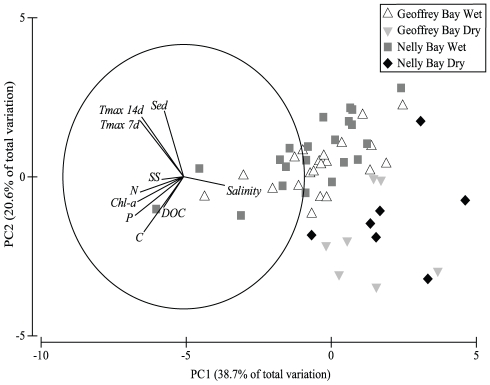
PCA of environmental variables. Principal coordinates analysis (PCA) of the nine measured environmental variables. Vector overlays represent multiple correlations between ordination axes and environmental parameters. PC1 is associated with particulate phosphorus (PP) and nitrogen (PN), chlorophyll-*a* (chl-*a*) and salinity whereas PC2 is associated mainly with sedimentation (Sed), maximum temperature 14 days preceding and including the sampling date (Tmax 14d) maximum temperature 7 days preceding and including the sampling date (Tmax 7d) and particulate organic carbon (POC). Together PCA1 and PCA2 axes capture 59.3% of the total variation of numbers of coral colonies with AN.

The exploratory multivariate ordination of the nine environmental parameters in a principal coordinates analysis (PCA) showed that some of the environmental variables were highly correlated (Fig. 5). The first principal component, PC1, was associated with water column concentrations of PP (eigenvalue −0.402), chlorophyll-*a* (chl-*a*; −0.382), PN (−0.361) and salinity (0.341). PC2 was associated with sedimentation (0.498), maximum temperature 14 days preceding and including the sampling date (Tmax 14d; 0.452), maximum temperature 7 days preceding and including the sampling date (Tmax 7d; 0.423) and POC (−0.417) (Fig. 5). Together, PC1 and PC2 explained 59.3% of the total variation in the data.

### (c) PLS regression

In the initial Partial Least Squares (PLS) regression model containing all nine environmental variables, many of the variables had low regression coefficients (R^2^) and hence were not good predictors of the variance in the number of AN cases. These predictors were therefore removed from the model in a stepwise manner.

The variance associated with AN abundance in the PLS model conducted using the combined datasets from both bays was significantly related to salinity, POC and Tmax 7d (F_3,167_ = 111.88, p<0.0001). More than 95% of the variance in the model was found within its first component. The model explained 67% of the variance within the first three components (Global R^2^ = 0.67). Following cross-validation, the model for both bays still explained 63% of the variation (predicted Global R^2^ = 0.63). When the model was broken down to regression coefficients, AN abundance was negatively correlated with salinity (R^2^ = −0.6) and positively correlated with POC (R^2^ = 0.32) and Tmax 7d (R^2^ = 0.08) (Table 2). The Durbin-Watson statistic was 1.39 indicating that there was no autocorrelation in the data set.

**Table 2:** Results of the PLS regression for the whole data set.

	ANOVA				Model Selection and Validation			Disease Predictors	
**All data**	P-value	F	D.F.	Component	Global R^2^	Predicted Global R^2^	Salinity	POC	Tmax 7d
PLS ANOVA	<0.0001	111.88	167	1	0.65	0.62	−0.6		
PLS Model				2	0.67	0.63		0.32	
				3	0.67	0.63			0.08

Results of the final model for the combined data set of AN versus the most important disease predictors: salinity, particulate organic carbon (POC) and maximum temperature 7 days preceding and including the sampling date (Tmax 7d). The PLS model was highly significant with its first three components explaining 74% of the variation. Following cross-validation the model still explained 36% of the variation.

## Discussion

As coastal human populations continue to increase, nutrients, terrigenous silt, pollutants and even pathogens themselves can be released to nearshore waters [35]. While the link between anthropogenic stress and disease susceptibility is currently poorly understood, it is thought that coral disease is facilitated by a decrease in water quality [22]. Evidence of this exists from the Caribbean [22], [23], [36] and the Philippines [20] and suggests that anthropogenic stressors and coral disease are linked in complex ways [35].

The present study documents a direct correlation between temporal coral disease dynamics and environmental parameters associated with water quality. The summer outbreaks of atramentous necrosis corresponded to minima in seawater salinity but maxima in all other water quality parameters investigated. The disease was strongly and negatively correlated with salinity and positively correlated with seawater concentrations of particulate organic carbon (POC).

The more pronounced AN outbreak in the summer of 2009 than in 2008 may be attributed to a greater terrestrial runoff caused by higher rainfall and higher values for environmental parameters (lower for salinity) preceding the outbreak in 2009. This may have lead to increased stress on corals that may have reduced their immune responses, and/or increased virulence of pathogen(s) causing the disease. Decreased resistance of the host coral caused by adverse environmental conditions may also increase opportunistic diseases [37]. Intense wet seasons may become more common in the future since strong rainfall events are a likely scenario associated with climate change [38]. Rainfall may be more variable from month to month, with longer dry spells and possibly with an increased frequency of disturbance events such as flooding rains and cyclones [39]–[42] which may lead to drastic changes in inshore salinity levels.

While black band disease prevalence showed no relationship with salinity in the Caribbean [43], the results of the present study indicate that low salinity promoted AN outbreaks. In 2009, salinity decreased in one month rapidly from above 30 to 20 in GB and to 19 in NB. Salinity measurements only commenced during the 2008 disease peak once this parameter was identified as a likely driver of AN, therefore lower salinity values may have occurred in the preceding weeks. Low salinity adversely affects corals [44] by harming coral fertilization [45], by affecting the processes of photosystem II [46] and, in extreme cases, by causing a breakdown in coral-zooxanthellae symbiosis leading to coral bleaching [47].

The role of POC in coral infections has not been investigated previously; however, dissolved organic carbon (DOC) has been linked to coral disease [24], [48]. High levels of DOC increased the growth rates of microbes and DOC was more detrimental to coral health than nutrients (nitrate, phosphate, ammonia) [24]. The authors suggested that there was a disruption in the balance between the coral and its associated microbes, subsequently shifting the microbial consortia resulting in disease. DOC compounds released by macroalgae were found to increase microbial activity [48]. These findings suggest that increasing DOC levels associated with inputs of sewage and organic waste from coastal development could contribute to the high incidence of disease on highly polluted reefs [24]. In the present study, the higher summer dissolved organic carbon (DOC) values could have facilitated AN infections by increasing the growth rates of microbes. High values of DOC and POC at the study sites were likely associated with increased pelagic and benthic primary production in the water after increased nutrient inputs following heavy rainfall and runoff [49], [50].

In the future, a combination of sea-level rise and an increase in rainfall due to climate change [38] could synergistically alter runoff and salinity in coastal ecosystems [51]. The duration and intensity of the rainy season will be important factors in determining the stress caused to corals since a long duration could lead to chronic stress. Previous studies have concluded that chronic stressors may be more harmful to corals than acute stressors but their impact will depend on the period of exposure to those stressors [52]. With the increasing probability of strong rainfall events leading to increased runoff in the future, both low salinity and high POC levels may lead to serious impacts on inshore reefs. It is likely that most inshore reefs of the GBR are heavily impacted by runoff during the wet season and that other reefs with high *Montipora* cover may experience similar outbreaks of AN like the ones on Magnetic Island. To date, no studies of how runoff impacts other coral diseases and other coral genera have been undertaken on the GBR and investigating this should be a priority in coral disease research.

Previous studies have identified clear seasonal patterns related particularly to warm temperatures for other coral diseases, including white syndrome (WS) [14], [53], black band disease (BBD) [16], and ulcerative white spots [54] on the GBR, and aspergillosis [13], white pox [21] and BBD [43] in the Caribbean. Earlier studies on AN [32] documented an outbreak on reefs around Magnetic Island when the water temperature was higher than 31.5°C. In the present study, the water temperature was 31.7°C during the outbreak of 2009. In the month preceding the outbreaks (Fig. 4, Table 1), temperature increased more in 2009 than in 2008, which may also have contributed to the larger number of recorded disease cases in 2009.

It is important to recognize that ecological responses to multiple interacting environmental variables are highly dynamic and rarely linear across both space and time with natural processes characterized by thresholds and limiting functions [55], [56]. Temperature, although not showing a strong correlation in the PLS regression, is still likely to contribute to disease abundance but the response may not be linear. The PCA analysis revealed that maximum temperature 7 (Tmax 7d) and 14 days (Tmax 14d) preceding and including sampling dates explained some of the variability in disease abundance (Fig. 4). Temperature preceding the outbreak may be important in AN dynamics as the PLS regression based on the whole dataset revealed that, although having a low regression coefficient, Tmax 7d was the third most significant environmental variable after salinity and POC (Table 2). The rate of temperature change is potentially important in AN dynamics and merits further investigations [57]. Further studies should also measure the duration of warm and cold periods that may impact AN dynamics to better understand the role of temperature in AN dynamics. Periods of hot and cold seawater, or hot and cold ‘snaps’, had an effect on patterns of white syndromes (WS) on the GBR, with most outbreaks occurring after mild winters and during hot summers [58].

In the present study, the highest water column nutrient concentrations were measured during the wet season. This agrees with previous studies that found that most water quality parameters other than salinity are higher during the wet season in the inshore GBR lagoon, when water quality conditions can change abruptly and nutrient concentrations increase dramatically for short periods following major disturbance events (cyclonic mixing, river flood plumes) [59]. Flood plumes are the main delivery mechanism for nutrients (in dissolved and particulate form) and suspended sediments to GBR coastal waters, with concentrations 10 to 400 times higher than in non-flood conditions [60], [61]. The coastal zone of the Burdekin region, where Magnetic Island is located, had the highest values of PN, PP and SS and second highest for chl-*a* of the whole GBR [62]. The Burdekin River exports very large amounts of sediment and associated nutrients during large floods [25] and significantly affects the water quality of Magnetic Island [63]–[65], together with local runoff from the island itself and from smaller rivers in the vicinity.

Sediments may not only be a cause of physical stress to corals but may also act as a pathogen reservoir [23]. For example, significantly higher sedimentation rates were found on sites with black band disease than on sites with no signs of disease in the Caribbean [23]. The highest sedimentation rates were found during the summer in the present study when AN abundance was high. By stressing corals, sediments may make the corals more susceptible to infections by microbial pathogens and may also act as disease reservoirs [23]. Fine sediment often settles on Magnetic Island reefs during periods of calm weather, and can result in smothering and tissue mortality of corals if the sediment is not re-suspended during rough weather or removed by the coral itself [28], [66], [67]. It is possible that the sediments act as pathogen reservoirs on Magnetic Island, however this was beyond the scope of the present study.

Coastal areas are globally under increasing pressure by human population growth, intensifying land use, urban and industrial development. However, previous studies on terrestrial influences on coral disease prevalence in the Indo-Pacific have not included direct measurements of water quality [20], [68]. Our study highlights a previously unrecognized adverse effect of land runoff on the health of key reef-building corals: the promotion of coral disease. The findings of this study are of wide importance because improving water quality in areas affected by runoff is one of the few management options that will enhance reef resilience in the face of climate change [9], [69].

## Methods

A research permit for this study was provided by the Great Barrier Reef Marine Park Authority (GBRMPA).

### (a) Study site and assessment of disease dynamics

The study sites were located in two adjacent bays, Nelly Bay and Geoffrey Bay, on the south-eastern side of Magnetic Island (19°S, 147°E), which is situated within the inner shelf region of the Great Barrier Reef. Both bays have fringing coral reefs and are similar in shape, physical structure, and hydrodynamic setting [70]. The study was conducted between December 2007 and December 2009. Sampling was conducted every 2 weeks in the austral summer (Nov-Apr) and once a month in the winter (May-Oct). Increased sampling frequency in summer was based on the hypothesis that AN increases with warm water temperatures [32], [33]. Disease dynamics were assessed in three permanent 5×5 m quadrats at 3–5 m depth at each site. Coral colonies demonstrating signs of AN [33] were tagged with numbered plastic tags attached to cable ties. Visual surveys were able to clearly distinguish the four stages in the development of AN lesions described above, although the last two stages were combined because they generally occur simultaneously. Thus, in the present study, the disease stages were referred to as: AN1 ( = stage 1), AN2 ( = stage 2), AN3 ( = stages 3 and 4), and S ( = disease progression stopped).

The diseased corals were all colonies of *Montipora aequituberculata,* which was the most prevalent species of *Montipora* in the quadrats. New disease cases (disease incidence) were counted and tagged in each plot during each survey. New AN infections, in addition to both lesion progression and cessation, were monitored on individual colonies to elucidate spatiotemporal patterns in disease dynamics. Due to logistical constraints, Geoffrey Bay was not sampled in February 2008.

### (b) Environmental parameters

At each sampling occasion, two replicate water samples were collected in 1-L plastic bottles 1 m above the coral and on opposite sides of the quadrats for the analysis of concentrations of: dissolved organic carbon (DOC), chlorophyll *a* (chl-*a)*, particulate organic carbon (POC), particulate nitrogen (PN), particulate phosphorus (PP) and suspended solids (SS). Due to logistical reasons, only two replicate water samples were used. Three physical variables were also measured, i.e. salinity, temperature and sedimentation.

DOC samples were filtered immediately through a 0.45 µm syringe filter (Sartorius MiniSart N) into acid-washed, screw-cap plastic test tubes. Samples were acidified by adding 100 µl of AR-grade hydrochloric acid (32%) and stored at 4°C until analysis. The concentrations were measured by high temperature combustion (680°C), using a Shimadzu Total Organic Carbon TOC-5000A carbon analyser. Prior to analysis, CO_2_ remaining in the sample water was removed by sparging with O_2_ carrier gas [71].

For the chl-*a* analysis, a 100 ml sub-sample was filtered immediately onto a 25 mm pre-combusted glass fibre filter (Whatman GF/F). Filters were wrapped in pre-combusted aluminium foil envelopes and stored at −18°C until analysis. Chl-*a* concentrations were measured fluorometrically using a Turner Designs 10AU fluorometer after grinding the filters in 90% acetone [72].

For analyses of POC, PN and PP, sub-samples of 250 ml were filtered onto 25 mm pre-combusted glass fibre filters (Whatman GF/F) and stored at −18°C. PN was determined by high temperature combustion using an ANTEK 9000 NS Nitrogen Analyser [26]. PP was determined spectrophotometrically as inorganic P (PO_4_, [73] after digestion in 5% potassium persulphate [72]. POC was determined by high temperature combustion (950°C) using a Shimadzu Total Organic Carbon TOC-V carbon analyser fitted with a Solid Sample Module SSM-5000A after acidification with concentrated phosphoric acid [71]. Inorganic C on the filters (e.g. CaCO_3_) was removed by acidification of the sample with 2M hydrochloric acid, the filter introduced into the sample oven (950°C), purged of atmospheric CO_2_ and the remaining organic carbon combusted in an oxygen stream and quantified by an infrared gas analyser.

Sub-samples for suspended solids (SS) were collected by filtering 1000 mL of water onto pre-weighed, 0.4 µm, polycarbonate filters (47 mm diameter, GE Water & Process Technologies), and SS concentrations were determined gravimetrically from the weight difference between loaded and unloaded filters after drying overnight at 60°C [71].

Salinity was measured at each sampling occasion with a hand-held refractometer (r^2^ Mini, Reichert GmbH, Germany). Temperature was measured using a temperature logger (ODYSSEY data recording systems, Christchurch, New Zealand) attached underneath a sediment trap in both Nelly and Geoffrey bays. It was retrieved and downloaded approximately every 2 months. Temperature data from sensors were combined with data collected by the Australian Institute of Marine Science (AIMS) sea surface temperature monitoring program in the same two bays (data available at http://www.aims.gov.au). Maximum temperatures were calculated for the periods of 7 and 14 days up to and including the sampling date.

Two sediment traps (40 cm high with a diameter of 10 cm) were deployed 10 m apart close to the permanent 5×5 m quadrats at each site. Traps were collected at every second sampling occasion in the winter and on each occasion in the summer. After decanting the seawater, the sediment was carefully transferred from the trap into a polycarbonate sample jar using a wash bottle with seawater. Salt in the samples was removed by adding distilled water, gently mixing the sediment and discarding the supernatant after the sediment had settled for a short time. This was repeated three times. Sediment samples were dried at 60°C for at least 3 days prior to determining their dry weight. The ash-free dry weight (AFDW) of the sediment was determined after combusting the sample at 450°C in a muffle furnace for 24 hours. The AFDW was used as a coarse measure of the organic content of the sediment.

Rainfall data for Townsville were obtained from the Australian Bureau of Meteorology web site (http://www.bom.gov.au).

### (c) Statistical analyses

Relationships between environmental parameters measured at the field sites were explored using a principal coordinates analysis (PCA) in PRIMER version 6.1.10 [75]. PCA results were summarized in a bi-plot containing the distribution of environmental parameters in two-dimensional space and their correlations with the PCA axes.

To investigate potential relationships between environmental parameters and AN prevalence in more detail, a Partial Least Squares (PLS) regression model was developed in Minitab. This technique is an extension of multiple regression analysis, in which the effects of linear combinations of several predictors on a response variable (or multiple response variables) are analyzed in a stepwise manner to remove descriptive variables that do not contribute to the model. PLS regression is particularly suited to cases in which the matrix of predictors has more variables than observations, or when there is multi-collinearity among variables [76]. This technique was first used in analytical chemistry and has been applied to analyses of ecological data since the late 1990s [76] and in recent publications [77]. Monthly disease prevalence data were analysed against maximum temperatures for the periods of 7 and 14 days up to and including the sampling date, assuming a time lag in the corals' response to changing environmental parameters. When observations were missing for temperature, sedimentation, POC, PN, PP, chl-a, DOC and SS, mean values of data before and after the missing data point were used to fill data gaps to be able to run the PLS regression. The PLS analysis calculates an analysis of variance table analogous to conventional multiple regression analysis, providing an overall assessment of the probability of statistical significance of the calculated PLS model. The PLS analysis also calculated a predicted residual sum of squares (PRESS) following cross-validation. This allowed for the calculation of a predicted Global R^2^ value in addition to a conventional Global R^2^, hence determining the predictive power of the observed relationship. A predicted Global R^2^ value lower than the conventional Global R^2^ indicates that the model is dependent upon only a few observations and does not have good predictive power.
