# Correction: Seasonal Rainfall and Runoff Promote Coral Disease on an Inshore Reef

**DOI:** 10.1371/annotation/365162ee-3718-44ce-b2e9-88302d5e0801

**Published:** 2011-03-08

**Authors:** Jessica Haapkylä, Richard K. F. Unsworth, Mike Flavell, David G. Bourne, Britta Schaffelke, Bette L. Willis

References 60 and 71 contained errors. The correct references are: "60. Rasheed MA, Unsworth RKF (2011) Long-term climate associated dynamics of a tropical seagrass meadow: implications for the future. Marine Ecology Progress Series. 422: 93-103" and "71. Unsworth RKF, Cullen LC (2010) Recognising the necessity for Indo-Pacific seagrass conservation. Conservation Letters 3: 63-73"

